# Inactivation of Osteoblast PKC Signaling Reduces Cortical Bone Mass and Density and Aggravates Renal Osteodystrophy in Mice with Chronic Kidney Disease on High Phosphate Diet

**DOI:** 10.3390/ijms23126404

**Published:** 2022-06-08

**Authors:** Ariane Zaloszyc, Philippe Choquet, Amira Sayeh, Maria Bartosova, Betti Schaefer, Ulrike Huegel, Gaëlle Aubertin-Kirch, Christopher Healy, François Severac, Sébastien Rizzo, Georges Boivin, Franz Schaefer, Michel Fischbach, Justine Bacchetta, Seiamak Bahram, Claus Peter Schmitt

**Affiliations:** 1 Service de Pédiatrie 1, Hôpital de Hautepierre, Hôpitaux Universitaires de Strasbourg, 67098 Strasbourg, France; fischbam@gmail.com; 2 Imagerie Préclinique—UF6237, Pôle d’Imagerie, Hôpitaux Universitaires de Strasbourg, 67098 Strasbourg, France; pchoquet@unistra.fr (P.C.); amira.sayeh@chru-strasbourg.fr (A.S.); 3 OMICARE, Centre de Recherche d’Immunologie et d’Hématologie, Fédération Hospitalo-Universitaire, 67085 Strasbourg, France; siamak@unistra.fr; 4 INSERM UMR_S 1109, Immuno-Rhumatologie Moléculaire, Centre de Recherche d’Immunologie et d’Hématologie, 67085 Strasbourg, France; 5 Center for Pediatric and Adolescent Medicine, Division of Pediatric Nephrology, University of Heidelberg, 69120 Heidelberg, Germany; maria.bartosova@med.uni-heidelberg.de (M.B.); betti.schaefer@med.uni-heidelberg.de (B.S.); uhuegel2017@gmail.com (U.H.); franz.schaefer@med.uni-heidelberg.de (F.S.); clauspeter.schmitt@med.uni-heidelberg.de (C.P.S.); 6 Pôle de Médecine et Chirurgie Bucco-Dentaires, Hôpitaux Universitaires de Strasbourg, 67091 Strasbourg, France; 7 Medical Image Analysis Center (MIAC AG), 4051 Basel, Switzerland; gaelle_aubertin@hotmail.com; 8 Department of Craniofacial Development and Stem Cell Biology, Dental Institute, King’s College London, London SE1 9RT, UK; chealy@chealy.plus.com; 9 Groupe Méthodes en Recherche Clinique (GMRC), Hôpital Civil, Hôpitaux Universitaires de Strasbourg, 67000 Strasbourg, France; francois.severac@chru-strasbourg.fr; 10 INSERM, UMR 1033, Université de Lyon, 69372 Lyon, France; sebastien.rizzo@chu-lyon.fr (S.R.); georges.boivin@univ-lyon1.fr (G.B.); justine.bacchetta@univ-lyon1.fr (J.B.); 11 Centre de Référence des Maladies Rénales Rares, Centre de Références des Maladies Rares du Calcium et du Phosphore, Hospices Civils de Lyon, 69500 Lyon, France; 12 Plateforme GENOMAX, Laboratoire d’Immuno-Rhumatologie Moléculaire, INSERM UMR_S1109, LabEx Transplantex, Centre de Recherche d’Immunologie et d’Hématologie, Faculté de Médecine, FMTS, Université de Strasbourg, 67085 Strasbourg, France; 13 INSERM Franco-Japanese Nextgen HLA Laboratory, 67085 Strasbourg, France; 14 Laboratoire Central d’Immunologie, Plateau Technique de Biologie, Pôle de Biologie, Nouvel Hôpital Civil, 67091 Strasbourg, France

**Keywords:** preclinical studies, parathyroid related disorder, CKD-MBD, bone scintigraphy, bone μCT

## Abstract

Chronic kidney disease (CKD) frequently leads to hyperphosphatemia and hyperparathyroidism, mineral bone disorder (CKD-MBD), ectopic calcifications and cardiovascular mortality. PTH activates the osteoanabolic Gα_s_/PKA and the Gα_q/11_/PKC pathways in osteoblasts, the specific impact of the latter in CKD-MBD is unknown. We generated osteoblast specific Gα_q/11_ knockout (KO) mice and established CKD-MBD by subtotal nephrectomy and dietary phosphate load. Bone morphology was assessed by micro-CT, osteoblast function by bone planar scintigraphy at week 10 and 22 and by histomorphometry. Osteoblasts isolated from Gα_q/11_ KO mice increased cAMP but not IP3 in response to PTH 1-34, demonstrating the specific KO of the PKC signaling pathway. Osteoblast specific Gα_q/11_ KO mice exhibited increased serum calcium and reduced bone cortical thickness and mineral density at 24 weeks. CKD Gα_q/11_ KO mice had similar bone morphology compared to WT, while CKD Gα_q/11_-KO on high phosphate diet developed decreased metaphyseal and diaphyseal cortical thickness and area, as well as a reduction in trabecular number. Gα_q/11_-KO increased bone scintigraphic tracer uptake at week 10 and mitigated tracer uptake in CKD mice at week 22. Histological bone parameters indicated similar trends. Gα_q/11_-KO in osteoblast modulates calcium homeostasis, bone formation rate, bone morphometry, and bone mineral density. In CKD and high dietary phosphate intake, osteoblast Gα_q/11_/PKC KO further aggravates mineral bone disease.

## 1. Introduction

Chronic kidney disease (CKD) is highly prevalent and inevitably associated with the disorder of mineral and bone metabolism, CKD-MBD [1,2,3,4]. The pathophysiological interplay of CKD and mineral bone disorder [5] includes abnormalities of calcium, phosphorus, parathyroid hormone, fibroblast growth factor 23, and vitamin D. It results in osteodystrophy, an increased fracture risk and extra-osseous calcifications, including vascular calcifications and thus contributes to the exceedingly high cardiovascular morbidity and mortality in patients with CKD [6,7]. The histological changes of bone are driven by the degree of CKD and the associated alterations in hormone and mineral metabolism, as well as by the therapeutic interventions to control CKD-MBD. Secondary hyperparathyroidism (HPT) results in high bone turnover with increased osteoblast, osteoclast and osteocyte numbers, and osteitis fibrosis. Overshooting therapy with vitamin D sterols and calcium results in adynamic osteodystrophy, increasing the risks of hypercalcemia and extra-osseus calcifications [3,4,8]. Only a minority of patients with CKD have a largely normal bone morphology [9]. Even in the initial stages of CKD, bone impairment is prevalent and fracture risk is increased [10,11,12] despite a phosphate reduced diet and various pharmaceutical measures including native and active vitamin D, oral phosphate binders, and calcimimetic agents [13]. Therefore, novel therapeutic approaches are needed.

PTH plays a crucial role in bone, calcium, and phosphate homeostasis. In bone, PTH binds to the class B PTH/PTH-related peptide receptor, PTH1R, which belongs to the G protein-coupled receptors class II family and activates G proteins Gα_s_ and Gα_q/11_ [14,15]. Gα_s_ activates the adenylyl cyclase and increases intracellular cyclic AMP (cAMP), leading to protein kinase A activation, which phosphorylates target proteins. Gα_q/11_ activates phospholipase C, which leads to the generation of inositol 1,4,5-triphosphate (IP3), and diacylglycerol [16], an increase in intracellular calcium and consecutive activation of the PKC signaling pathway [14,17]. The effects of PKA activation and inactivation have been described in mice and in human genetic disorders, and demonstrated the role of PKA in the response of bone to PTH [18,19,20,21,22,23]. cAMP/PKA mediates the majority of PTH effects, including bone formation and bone resorption by increasing the expression of RANKL [18,19,24]. However, the role of PKC signaling is less clear [24]. In mice, genetic activation of bone Gα_q_ or Gα_11_ results in severe osteoporosis with a decrease of both trabecular and cortical bone volume, and in case of Gα_q,_ activation in dwarfism. The anabolic response to intermittent PTH treatment is absent [25,26]. Mice with a genetic variant of the PTH1R blocking PKC activation and maintaining PKA activation (DSEL mice) have a decreased trabecular bone volume but develop less bone alterations in response to continuous PTH administration or a low calcium diet, as compared to WT mice [27,28]. Ogata et al. established an osteoblastic specific knockout Gα_q/11_ mouse model, which yielded no alterations in bone structure at 8 weeks of life, but an increase anabolic response to intermittent PTH treatment [29]. In contrast, the intermittent administration of an analog of PTH, which was recently identified to activate PKC but not PLC and cAMP/PKA signaling, lead to an osteoanabolic effect [24], suggesting a selective, significant osteoanabolic action of PKC activation.

In view of the major clinical impact of CKD-MBD and the lack of specific therapeutic approaches improving bone disease, we studied the role of PKC modulation in mice with CKD. Therefore, we established an osteoblast specific Gα_q/11_ knockout, blocking the PKC signaling pathway in mice with normal renal function, CKD, high phosphate diet, and the combination of both. 

## 2. Results

### 2.1. Experimental Results

#### 2.1.1. Osteoblast Gα_q/11_ Expression and PTH Response

**Figure 1:**
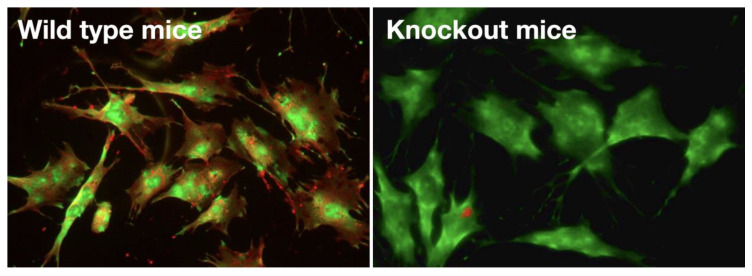
Immunostaining of PTH1R and Gα_q/11_. Osteoblasts isolated from bone of wild type mice (**left** figure) and from bone of Gα_q/11_ knockout mice incubated with PTH1-34 for one hour, were stained for PTH1R (green) and Gα_q/11_ protein (i red). On the **right**, osteoblasts from Gα_q/11_ -KO mice express PTH1R but not Gα_q/11_. Total magnification: ×630.

Immunostainings demonstrated the absence of Gα_q/11_ protein in PTH stimulated osteoblasts isolated from Gα_q/11_ KO mice and the distinct abundance in osteoblasts from WT littermates (Figure 1).

**Figure 2:**
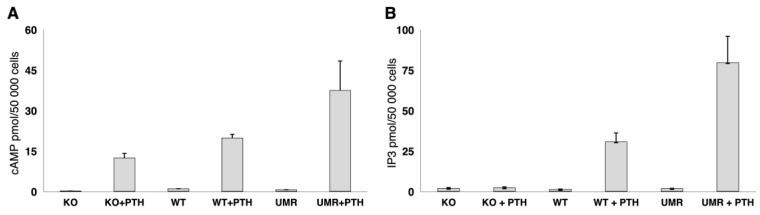
(**A**) cAMP and (**B**) IP3 response to PTH1-34 in WT, in Gα_q/11_ knockout osteoblasts and in immortalized UMR osteoblasts WT and Gα_q/11_-KO osteoblasts and UMR osteoblasts were stimulated with PTH 1-34 for 15 min and cellular cAMP and IP3 response was quantified. While PKA mediated cAMP response was still present in the Gα_q/11_-KO osteoblasts, the Gα_q/11_/PKC mediated induction of IP3 was abolished in the Gα_q/11_-KO osteoblasts.KO = Gα_q/11_ knockout osteoblasts; WT = wild type osteoblasts; UMR = immortalized osteoblast-like UMR106-01 cells; +PTH = incubation with PTH1-34.

Stimulation of the Gα_q/11_ KO osteoblasts with PTH increased PKA signalling dependent medium cAMP concentrations, whereas PKC signalling dependent IP3 stimulation was absent in the osteoblasts from KO mice. Osteoblasts from WT mice and immortalized osteoblast-like UMR106-01 cells have PTH induced cAMP and IP3 responses, providing evidence for the functional KO of PKC signalling in response to PTH stimulation in the Gα_q/11-_ KO osteoblasts (Figure 2A,B).

#### 2.1.2. Body Weight, Mortality, and Serum Biochemistry

- Body Weight:

At week 10, baseline body weight was similar in the eight subgroups of mice; mean body weight was 24.3 ± 2.3 g. Baseline serum calcium concentrations were increased in Gα_q/11_ KO compared to WT mice (2.62 ± 0.1 versus 2.37 ± 0.1 mmol/L; *p* < 0.0001), whereas serum creatinine, urea, albumin, and phosphate were comparable.

At time of sacrifice (week 24), body weight was not different in Gα_q/11_-KO as compared to WT mice, and not different in Gα_q/11_-KO_CKD_ versus WT_CKD_ mice. Gα_q/11_-KO_HP_ and Gα_q/11_-KO_CKD-HP_ had a lower body weight compared to respective WT_HP_ and WT_CKD-HP_ mice (*p* = 0.033 and *p* = 0.010). Thus, the genetic KO of Gα_q/11_ reduced body weight in the context of high phosphate diet, but not in mice with CKD and in mice with normal renal function on standard diet (Table 1).

- Mortality:

Overall mortality rate was 20%: 10% in WT, 11% in WT_HP_ mice, 30% in Gα_q/11_-KO, 20% in Gα_q/11_-KO_HP_ mice, 17% in WT_CKD_, 11% in WT_CKD-HP_, 8% in Gα_q/11_-KO_CKD_, and 43% in Gα_q/11_-KO_CKD-HP_.

- Biochemistry:

Serum biochemistry at sacrifice was comparable between groups, except for CKD animals, which developed a marked increase in serum creatinine, urea, and phosphate concentrations. Serum phosphate was also increased in the non-CKD WT_HP_ and Gα_q/11_-KO_HP_ mice compared to respective WT and Gα_q/11_-KO mice on standard diet (*p* = 0.003 and *p* = 0.02, respectively) (Table 1). Serum calcium was increased in Gα_q/11_-KO_HP_ mice compared to respective WT_HP_ mice (*p* < 0.001). CKD and high phosphate diet both induced hyperparathyroidism, especially in mice with combined CKD and high phosphate diet. Serum PTH was increased in CKD mice, with dietary phosphate load, and the combination of both by 4-, 2-, and 24-fold compared to controls, the genotype had no effect (Table 1).

#### 2.1.3. Bone Morphology (µCT)

At week 24, femur length was similar in all groups (Table 2). CKD decreased femur cortical and trabecular total mineral density (TMD) in WT mice (*p* = 0.0002/0.001 for diaphyseal/metaphyseal cortical TMD and *p* = 0.05 for trabecular TMD). High phosphate diet in WT mice with normal renal function decreased trabecular TMD (*p* = 0.01/0.02). In WT_CKD-HP_ mice cortical area but not TMD was increased compared to WT mice (*p* = 0.04 for diaphysis and *p* = 0.05 for metaphysis). 

**Figure 3:**
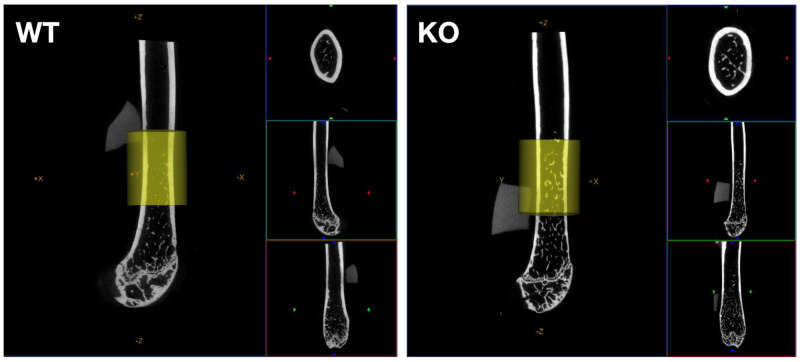
Micro-CT views of mouse femurs (WT on the **left** and KO on the **right**) in 3 perpendicular planes of section. A yellow ROI placed on the magnified long axis section delineates the area in which cortical thickness was measured. Cortical thickness was decreased in Gα_q/11_-KO mice compared to WT mice at Week 24.

Mice with osteoblast Gα_q/11_-KO had decreased cortical TMD both in the diaphysis and metaphysis (*p* = 0.0004/*p* = 0.04), as well as a reduced cortical thickness in the diaphysis (*p* = 0.006) compared to WT mice (Figure 3, Table 2), while the Gα_q/11_-KO had no effect in CKD mice. In contrast, in CKD mice on high phosphate diet, the osteoblast Gα_q/11_-KO resulted in a lower diaphyseal and metaphyseal cortical thickness and area compared to WT_CKD-HP_ mice (*p* = 0.0001/0.01/0.001/0.001); the trabecular number was lower and the trabecular spacing increased (*p* = 0.008/0.003). In non-CKD mice on high phosphate diet, the Gα_q/11_-KO mice decreased metaphyseal cortical area compared to WT_HP_ mice (*p* = 0.04) (*p* = 0.006/0.05). Irrespective of the genotype, mice with CKD on high phosphate diet developed medullar and soft tissue ectopic calcifications.

#### 2.1.4. Bone Planar Scintigraphy Analysis

At week 10, Gα_q/11_-KO mice (*n* = 35) had an increased tracer uptake activity compared to WT mice (*p* = 0.002; Table 3).

At week 22, WT_CKD_ and WT_CKD-HP_ mice had an increased osteoblast activity compared to WT controls (*p* < 0.0001/0.007). Gα_q/11_-KO did not modify tracer uptake in mice with normal renal functional and standard diet, but mitigated the CKD induced increase in tracer uptake in the Gα_q/11_-KO_CKD_ mice compared to WT_CKD_ mice (*p* = 0.049). In mice on high phosphate diet and the combination of high phosphate diet and CKD, the Gα_q/11KO_ did not modify tracer uptake.

#### 2.1.5. Bone Histomorphometry

Bone histomorphometric indices of the left femurs did not differ with genotype, CKD status, or the diet. Only in mice with CKD and high phosphate diet BV/TV was increased in WT_CKD-HP_ and Gα_q/11_-KO_CKD-HP_ compared to WT and Gα_q/11_-KO mice (*p* = 0.032 and *p* = 0.009, respectively). The number of bone trabecula was increased in WT_CKD-HP_ as compared to WT mice (*p* = 0.02).

No statistically significant differences were found between WT and Gα_q/11_-KO mice with or without CKD in vertebra, but Gα_q/11_-KO mice tended to have higher values in formations parameters, i.e., osteoid parameters, osteoblast surface, and mineral apposition rate (Table 4).

## 3. Discussion

Chronic kidney disease is prevalent in the aging population, and on top of aging related loss of bone mass, CKD-MBD accelerates the loss of bone mass and strength, increases the risk of bone fracture rates, and results in physical disability [30]. Current therapeutic options are insufficient; hyperparathyroidism and hyperphosphatemia are prevalent [31,32]. A recent study demonstrated bone anabolic effects with selective PKC activation [24]. To assess the role of osteoblast PKC signaling in CKD and associated hyperphosphatemia, we now established a mouse model of osteoblast specific Gα_q/11_-KO. In these KO mice, PKA activation through PTH was maintained, while PKC activation was absent, as shown by the in vitro response of osteoblasts to PTH isolated from WT and Gα_q/11_-KO mice. These mice were exposed to CKD, high phosphate, and the combination of both. Femur bones were analyzed by micro-CT and planar scintigraphy, reflecting bone formation activity, and the vertebrae by quantitative histomorphometry. 

Under physiological conditions, Gα_q/11_ KO mice developed a lower cortical TMD and a lower cortical thickness until 24 weeks of life, despite increased phosphate tracer uptake at 10 weeks of age, and comparable bone formation activity at week 22 of life. Due to technical shortcomings, dynamic histomorphometry was assessed in only four groups (four mice per group) and suggested a trend towards increased bone formation parameters in mice with Gα_q/11_-KO, which aligns with the scintigraphic findings at week 10. Gα_q/11_ KO increased bone formation and turnover, while the decreased cortical bone thickness and bone mineral density in CT suggests increased cortical bone resorption by osteoclasts. To prove this notion, a respective bone histology analysis is needed, however could not be accomplished in this study due to insufficient preservation of the bones.

Our results differ from previous studies in mice with osteoblast specific PKC KO, in which no difference in cortical and trabecular structures were found. However, we studied the mice at the age at 24 weeks and not young mice at 12 weeks of life as done in the previous study [29]. It was found that 10-week -old DSEL mice predominantly developed trabecular alterations and only minor cortical changes [28,33]. PKC activation in osteoblasts had been shown to decrease trabecular and cortical bone volume [25,26,34]. Intermittent administration of a PTH analog, which was recently identified to selectively activate PKC, led to an osteoanabolic effect [24]. We now demonstrate that PKC KO in osteoblasts decreases cortical bone thickness and mineral density. 

In additions to previous studies, we now assessed the impact of Gα_q/11_-KO in mice with CKD. CKD increased bone tracer uptake, i.e., bone formation rate, a finding related to the associated and untreated hyperparathyroidism, which increases bone turn over in mice [35] and humans [36]. Consistent with this, cortical and trabecular bone TMD were decreased compared to WT control mice, which again was a finding in line with clinical findings in humans [37,38]. The Gα_q/11_-KO in CKD mice reduced tracer uptake on scintigraphy, i.e., bone formation rate, but none of the micro-CT derived bone parameters. In the presence of high phosphate intake, Gα_q/11_-KO, however, reduced cortical bone thickness, bons area, and trabecular bone number, i.e., further aggravated renal osteodystrophy of CKD. Of note, body weight and biochemical findings, including renal function parameters and serum PTH, did not differ between Gα_q/11_-KO and WT mice with CKD and high phosphate diet, indicating that the differences in bone morphology were specific to the functional changes caused by the Gα_q/11_-KO.

The deleterious effect of high phosphate intake on CKD-MBD and associated cardiovascular disease had repeatedly been demonstrated [39]. We now demonstrated that high phosphate intake worsened CKD-MBD induced renal osteodystrophy in case of osteoblast Gα_q/11_ KO. These mice developed pronounced hyperparathyroidism, increased serum calcium, and phosphate levels and consequently ectopic calcifications in bone marrow and soft tissues, i.e., a severe phenotype of CKD-MBD, known to be associated with poor prognosis in humans [40].

High phosphate diet also induced hyperparathyroidism in mice with normal renal function and a decrease in trabecular bone thickness and mineral density, untoward effects that were prevented by Gα_q/11_-KO in osteoblasts. Interestingly, in Gα_q/11_-KO mice the high phosphate diet even increased cortical thickness and mineral density.

Bone planar scintigraphy in CKD has limitations since knowledge on the impact of CKD on tracer bone uptake and elimination is limited. In humans, severe CKD leads to a decrease in scintigraphic tracer elimination, and uptake of the tracer in soft tissue, which may result in false estimates of bone disease. Late imaging has been proposed to solve this issue [41,42,43]. Still, bone radionuclide imaging has been shown in patients on dialysis to be correlated with histomorphometric dynamic parameters [44,45,46].

In summary, we demonstrated that osteoblast specific KO of Gα_q/11_-PKC signaling reduced bone mass and bone mineral density under physiological conditions. Renal osteodystrophy was aggravated in CKD mice with Gα_q/11_-KO together with high dietary phosphate intake, a condition frequently found in patients with CKD-MBD. Future studies should now address the putative therapeutic potential of specific modulation of specific osteoblast’s Gα_q/11_-protein signaling in mice with CKD and low and high phosphate diet, e.g., by novel PTH analog selectively activating PKC [24].

## 4. Materials and Methods

### 4.1. Mice

Male mice in a mixed C57BL6/N genetic background were used for the experiment. Animals were housed in standard conditions with individually ventilated, temperature-controlled cages (SealSafe 1291H, Tecniplast, Buguggiate, Italy) with environmental enrichment and 12 h light/dark cycle and *ad libitum* access to food and water. The osteoblast specific Gα_q/11_ knockout mice were generated by crossing global-deleted *Gnα_11_* mice (*Gnα_11_^−/−^*) with *Gnα_q_* gene flanked with loxP (*Gnα_q_^fl/fl^*) mice provided by Professor S. Offermanns [47] with specific osteocalcine promoter-*Cre* mice (*Oc-Cre^+/−^*) provided by Professor T.L.Clemens [48].

Intercrosses resulted in the generation of osteoblast specific double knockout mice (KO): *Oc-Cre^+/−^*; *Gnα_q_^fl/fl^*; *Gnα_11_^−/−^*. Control mice (WT) used in this study had *Oc^−/−^*; *Gnα_q_^fl/fl^* and *Gnα_11_^+/+^* genotype, respectively. Genomic DNA was isolated from the tails of the mice, according to the manufacturer’s instructions (Qiagen, Hilden, Germany) and the genetic background of the mice were assessed by standard PCR with specific primers and followed by electrophoresis. The presence or absence of *Gnα_11_* allele was determined using the upstream primer 5’-AGC ATG CTG TAA GAC CGT AG-3’ and downstream primer 5’-GCC CCT TGT ACA GAT GGC AG-3’ or the upstream primer 5′-GAC TAG TGA GACGTG CTA CTT CC-3′ and downstream primer 5’-CAG GGG TAG GTG ATG ATT GTGC-3,’ respectively. The presence of *Gnα_q_^fl/fl^* was determined using the following primers 5’-GAC TAG TGA GACGTG CTA CTT CC-3’ and 5’- CAG GGG TAG GTG ATG ATT GTGC-3′. The presence or absence of the *Oc-Cre* transgene was determined with Cre-specific primers (5’-CAA ATA GCC CTG GCA GAT-3’ and 5’-TGA AAG GGA CAT CTT CC-3’).

Gα_q/11_ KO and WT mice underwent a two-step 5/6 nephrectomy procedure to establish stable CKD (KO_CKD_ and WT_CKD_) as previously described [35,49,50,51]. Briefly, at the age of 10 weeks cortical electrocautery was applied to the right kidney, 2 weeks later the left kidney was removed. All other animals underwent sham surgery. CKD mice with a serum urea below 15 mmol/L were excluded from subsequent analyses.

Animals were randomized to either standard diet (WT, KO, KO_CKD_, and WT_CKD_), or high phosphate diet (0.5 or 1% phosphate, both from Altromin, Lage, Germany) (WT_HP_, KO_HP_, KO_CKD-HP_, and WT_CKD-HP_). Each group included 7 to 12 animals according to the genotype, CKD status, and diet. Body weight was recorded weekly for all mice. Blood samples were taken and bone planar scintigraphies were performed prior to surgery at 10 weeks of age, and at the end of the experiment at 22 weeks of age. Bone μCT was performed ex-vivo. To assess bone formation, calcein (Sigma, Saint-Louis, MO, USA 20 mg/kg) was injected subcutaneously 7 days and demeclocyclin (Sigma, Saint-Louis, MO, USA 50 mg/kg) two days before sacrifice. All surgical and imaging procedures were performed under gaseous anesthesia (isoflurane 5% for induction followed by isoflurane 1.5 to 2% pushed by air), on a warmed table and in imaging cells, respectively. At 24 weeks of age, mice were anesthetized with ketamine/xylazine (100 mg/kg, 20 mg/kg) and blood was collected via cardiac puncture. Through the same puncture needle NaCl 0.9% was infused followed by 4% formaldehyde to perfuse and fix the organs. After dissection, femurs and vertebras were immersed in 4% formaldehyde. All animal experiments were performed according to the EU regulations for animal experimentation. The project was approved by local authorities (Regierungspräsidium Karlsruhe, Karlsruhe, Germany; Nr.35-9185.81 G-12/12).

### 4.2. Cells Cultures

Osteoblast-like cells were isolated from long bones of 6 weeks old male mice (*n* = 4). After removal of the muscle and soft tissue, the bones were washed with sterile PBS and cut longitudinally into 1 mm long fragments. The fragments were incubated in 6-well plates (Falcon) in αMEM Medium (Lonza, Verviers, Belgium) supplemented with 10% FBS (Lonza, Verviers, Belgium), 1% penicillin/streptomycin (Biochrom, Berlin, Germany), and 5 mM glutamin (Biochrom, Berlin, Germany) for 21 days (humidified incubator, 37 °C, 5% CO_2_) to allow cell outgrowth. Media was exchanged weekly. After reaching confluency, the cells were trypsinized and sub-cultured in 1:4 ratio. For immunostaining, cells were plated onto coverslips in 24 well plates. The commercial rat osteosarcoma cell line (UMR 106, American Type Culture Collection ATCC, Manassas, VA, RRID:CVCL 3617) was used as control.

### 4.3. Immunostaining

For immunostaining, cells were fixed in 4% paraformaldehyde (PFA) for 10 min at room temperature, permeabilized (0.1% TritonX in PBS) for 3 min, washed 2 times with PBS, and blocked (1% horse serum in PBS) for 30 min at room temperature. 

Coverslips were incubated with anti-G protein alpha q/11 antibody (rabbit polyclonal, Abcam, 1/100 in PBS) overnight at 4 °C in a humified chamber. After three washing steps with PBS, secondary antibody (anti-rabbit, AlexaFluor 594, 1/1000 in PBS) was added for 1 h. If the second staining was performed, after washing with PBS, the slides were blocked again and incubated with PTHR1 antibody (mouse monoclonal, antibodies online, Aachen, Germany, 1/100 in PBS) for 1 h. After further washing steps, the secondary antibody was added for 1 h (anti-mouse, AlexaFluor 488, 1/1000). The coverslips were washed, mounted for immunofluorescence, and visualized with DMI 4000B microscope (Leica, Mannheim, Germany). 

### 4.4. Cyclic AMP and IP3 Assay

The cells were plated on coverslips (Marienfeld, Lauda-Königshofen, Germany) in 24-well plates (Falcon) and grown for 24 h to sub-confluence in the αMEM Medium (Lonza, Verviers, Belgium). After an overnight starvation, cells were incubated with and without PTH1-34 (Bachem CA, USA) in serum-free medium for 15 min for determination of cellular cAMP and IP3. For cyclicAMP and IP3 quantification, ELISA were used (Direct cAMP ELISA kit, Enzo Life Sciences, INC. Famingdale, USA and IP3 ELISA kit, Antibodies online, Aachen, Germany) according to manufacturer’s instructions.

### 4.5. Blood Chemistry Analysis

Blood was sampled under anesthesia, at 10 weeks of age, by puncture of the mandibular vein (maximal blood volume 0.2 mL) and again at sacrifice by intracardiac puncture and transferred to heparinized tubes. After centrifugation (1500 G for 1 min, Statspin, IDEXX, Westbrock, CT, USA), plasma was aliquoted and analyzed either on the same day or frozen at −80 °C. Plasma urea, creatinine, calcium, albumin, and phosphate were measured using a veterinary analyzer based on dry-slides technologies (VetTest, IDEXX, Westbrock, CT, USA). Intact PTH was measured by ELISA (Mouse PTH 1-84 Elisa kit, Immunotopics international, San Diego, CA, USA).

### 4.6. Micro-CT Analysis

Right femurs were scanned using a GE Locus SP (GE Healthcare, Waukesha, USA). The specimens were immobilized using cotton gauze, immersed in an alcohol solution, and scanned using an X-ray tube voltage of 80 kVp and a tube current of 80 μA. An aluminum filter (0.05 mm) was used to adjust the energy distribution of the X-ray source. The reconstructed imaging volume was made of cubic voxels of 7 × 7 × 7 μm^3^. Visualization and analysis were performed using MicroView (v 2.2, GE Healthcare, Waukesha, WI, USA), bone measurements using the Advanced Bone module. The specimens were characterized further by building three-dimensional isosurface renderings, generated by the marching cubes method. Cortical parameters were determined at 1700 μm distance of the distal growth plate (in diaphysis) and right over the growth plate (in metaphysis) using a cylindric region of interest (ROI) of 300 × 300 × 300 μm^3^ on which an automatic cortical threshold was applied. For trabecular parameters, a manual ROI was drawn adjacent to the endocortical boundary, right over the distal growth plate and with an extension of 180 μm. Trabecular separation, thickness, and numbers were calculated using 3D calculations (sphere fitting method). The plate–rod characteristics were estimated by the structure model index (SMI), and anisotropy degree was defined by the length of the longest divided by the shortest mean intercept length. Bone density was assessed by tissue mineral density.

### 4.7. Bone Planar Scintigraphy Analysis

Bone scintigraphy analyses were performed at week 10, prior to the surgical interventions, and again at week 22, i.e., 2 weeks prior to sacrifice. Osteoblast activity was quantified by bone planar scintigraphy as described elsewhere [52]. Following the manufacturer’s recommendations and good practices, 134 ± 24 MBq of ^99m^Tc-hydroxymethylene diphosphonate (HMDP) (CIS bio international, Gif-sur-Yvette, France) was prepared. The latter was administered under gaseous anesthesia through tail venous injection, after weighing animal. After 150 ± 53 min, pinhole whole-body acquisitions (15 min) with a window of 140 keV ± 10% were performed.

### 4.8. Histomorphometric Analysis

Decalcified left femurs were embedded in paraffin, and non-decalcified lumbar vertebrae were fixed, dehydrated, and embedded in methyl methacrylate. Longitudinal femur and transversal vertebra 8 µm-thick sections were obtained with a microtome (Polycut S, Leica, Heidelberg, Germany) and stained with a Goldner’s trichrome. ROI was selected from 250 to 1250 μm of the femur growth distal plate and analyzed together with all vertebrae. Identifications of osteoid and calcified tissue in vertebra were assessed using an automatic image analyzer (analyzer Morpho Expert, Explora Nova, La Rochelle, France) [53]. Dynamic parameter, i.e., mineral apposition rate was assessed from unstained 8 μm-thick sections examined under fluorescent light microscopy. Vertebra analysis was only performed in the four mice groups with standard diet.

### 4.9. Statistical Analysis

Results are expressed as mean ± standard deviation. Normality of the distribution was assessed by a Shapiro-Wilk test and a Q-Q plot. Gaussian continuous variables were analyzed using linear regression models including a three-way interaction between genotype, CKD, and diet. Comparisons between subgroups of interest (according to genotype but also influence of CKD, of diet, and the combination of CKD and diet) were performed using linear contrasts in the regression model. For non-gaussian variables we used Wilcoxon non-parametric test. Difference between groups were considered significant when *p* was <0.05. All the analyses were performed using R software. R Core Team. R: A language and environment for statistical computing. R Foundation for Statistical Computing, Vienna, Austria.

## Figures and Tables

**Table 1:** Body weight and serum biochemistry in the eight groups of mice at time of sacrifice (week 24). Comparisons were performed according to the genotype, the influence of CKD, of the diet, and the combination of CKD and diet. Mean values and standard deviations are given, superscripts indicate significant difference to the indicated group, findings in Gα_q/11_-KO mice significantly different to respective WT mice are given in bold (*p* < 0.05). WT = Wild type mice, KO = Gα_q/11_ knockout mice, WT_CKD_ = wild type mice with CKD, KO_CKD_ = Gα_q/11_ knockout mice with CKD, WT_HP_ = wild type mice with high phosphate diet, KO_HP_ = Gα_q/11_ knockout mice with high phosphate diet, WT_CKD-HP_ = wild type mice with CKD and high phosphate diet, KO_CKD-HP_ = Gα_q/11_ knockout mice with CKD and high phosphate diet.

	WT ^a^	KO ^b^	WT_CKD_^c^	KO_CKD_^d^	WT_HP_^e^	KO_HP_^f^	WT_CKD-HP_^g^	KO_CKD-HP_
Body weight (g)	30.6 ± 2.6	29.3 ± 2.1	27.4 ± 1.6	28.7 ± 2.2	33.2 ± 2.8 ^a^	**30.7 ± 3.4 ^e^**	28.6 ± 2.1	**25.0 ± 2.7 ^b,g^**
Creatinine (g/L)	0.70 ± 0.3	0.73 ± 0.2	2.01 ± 0.7 ^a^	2.67 ± 0.9 ^b^	1.08 ± 0.3	0.95 ± 0.3	3.06 ± 0.7 ^a^	2.69 ± 0.7 ^b^
Urea (mmol/L)	7.0 ± 0.6	6.9 ± 1.1	21.9 ± 3.1 ^a^	24.9 ± 6.4 ^b^	7.0 ± 1.0	7.7 ± 2.2	16.3 ± 2.3 ^a^	17.7 ± 3.0 ^b^
Albumine (g/L)	22.9 ± 2.1	20.6 ± 2.1	21.2 ± 2.6	21.5 ± 2.9	20.5 ± 4.4	20.1 ± 3.0	22.8 ± 2.2	21.9 ± 2.5
Calcium (mmol/L)	2.36 ± 0.1	2.37 ± 0.1	2.53 ± 0.1	2.64 ± 0.04 ^b^	2.10 ± 0.3 ^a^	**2.42 ± 0.2 ^e^**	2.57 ± 0.2 ^a^	2.64 ± 0.2 ^b^
Phosphate (mmol/L)	0.98 ± 0.2	1.03 ± 0.1	1.35 ± 0.3 ^a^	1.41 ± 0.4 ^b^	1.78 ± 0.3 ^a^	1.67 ± 0.2 ^b^	1.62 ± 0.08 ^a^	1.99 ± 0.5 ^b^
PTH (ng/L)	54 ± 37	70 ± 25	106 ± 95	173 ± 108 ^b^	229 ± 155 ^a^	293 ± 170 ^b^	1907 ± 1099 ^a^	1687 ± 789.1 ^b^

**Table 2:** Bone parameters of right femurs extracted from micro-CT acquisitions ex vivo. Comparisons were performed according to the genotype, the influence of CKD, of the diet, and the combination of CKD and diet. Superscript symbols indicate significant difference to the corresponding group (*p* < 0.05), findings in Gα_q/11_ KO mice significantly different to respective WT mice are given in bold. WT = wild type mice, KO = Gα_q/11_ knockout mice, WT_CKD_ = wild type mice with CKD, KO_CKD_ = Gα_q/11_ knockout mice with CKD, WT_HP_ = wild type mice with high phosphate diet, KO_HP_ = Gα_q/11_ knockout mice with high phosphate diet, WT_CKD-HP_ = wild type mice with CKD and high phosphate diet, KO_CKD-HP_ = Gα_q/11_ knockout mice with CKD and high phosphate diet, Ct = cortical, TMD = total mineral density, Th = thickness, Ar = area, BV = bone volume, TV = total volume, Tb = trabecular, N = number, Sp = spacing, DA = degree of anisotropy, SMI = structure model index.

	WT ^a^	KO ^b^	WT_CKD_^c^	KO_CKD_^d^	WT_HP_^e^	KO_HP_^f^	WT_CKD-HP_^g^	KO_CKD-HP_
	*n* = 6	*n* = 10	*n* = 9	*n* = 6	*n* = 8	*n* = 10	*n* = 7	*n* = 9
**Femoral length (mm)**	16.0 ± 0.2	15.9± 0.3	15.9 ± 0.2	16.1 ± 0.3	16.1 ± 0.2	15.8 ± 0.3	16.2 ± 0.1	15.7 ± 0.3
Cortical parameters (Diaphysis)								
**Ct.TMD (mg/cc)**	1269 ± 62	**1144 ± 79 ^a^**	1131 ± 47 ^a^	1159 ± 37	1202 ± 61	1202 ± 49 ^b^	1214 ± 94	1174 ± 70
**Ct.Th (μm)**	197 ± 141	**179 ± 15 ^a^**	191 ± 12	183 ± 13	195 ± 10	194 ± 17 ^b^	209 ± 105	179 ± 16 ^g^
**Ct.Ar (mm^2^)**	0.83 ± 0.06	0.77 ± 0.06	0.79 ± 0.04	0.80 ± 0.08	0.85 ± 0.06	0.82 ± 0.06	0.90 ± 0.07 ^a^	**0.73 ± 0.09 ^g^**
Cortical parameters (Metaphysis)								
**Ct.TMD (mg/cc)**	1142 ± 42	**1059 ± 59 ^a^**	993 ± 70 ^a^	1031 ± 48	1089 ± 60	1091 ± 27	1029 ± 122 ^a^	1067 ± 75
**Ct.Th (μm)**	158 ± 12	141 ± 17	159 ± 14	146 ± 5	171 ± 16	156 ± 17	169 ± 17	**148 ± 29 ^g^**
**Ct.Ar (mm2)**	0.76 ± 0.05	0.72 ± 0.07	0.80 ± 0.04	0.73 ± 0.03	0.89 ± 0.06 ^a^	**0.80 ± 0.06 ^e^**	0.86 ± 0.12 ^a^	**0.72 ± 0.16 ^g^**
Trabecular parameters								
**Tb TMD (mg/cc)**	765 ± 76	733 ± 90	690 ± 49 ^a^	712 ± 37	679 ± 54 ^a^	7116 ± 41	729 ± 102	754 ± 61
**BV/TV (%)**	9.1 ± 1.6	10.3 ± 2.7	9.6 ± 1.9	11.6 ± 2.6	7.6 ± 1.9	7.9 ± 1.7	9.5 ± 3.0	7.8 ± 4.4
**Tb.Th (μm)**	28.1 ± 3.1	27.4 ± 3.1	25.4 ± 2.2	28.4 ± 3.0	23.3 ± 3.6 ^a^	25.0 ± 2.3	26.2 ± 4.6	27.9 ± 4.9
**Tb.N (/mm)**	3.2 ± 0.3	3.7 ± 0.8	3.8 ± 0.8	4.1 ± 0.6	3.3 ± 0.7	3.2 ± 0.5	3.7 ± 11	**2.6 ± 1.0 ^b,g^**
**Tb.Sp (μm)**	283 ± 29	256 ± 62	248 ± 54	221 ± 38	295 ± 66	298 ± 51	272 ± 103	**394 ± 143 ^b,g^**
**DA**	1.20 ± 0.06	1.30 ± 0.15	1.15 ± 0.07	1.26 ± 0.10	1.18 ± 0.06	1.20 ± 0.06	1.22 ± 0.11	1.21 ± 0.06
**SMI**	2.32 ± 0.18	2.00 ± 0.31	2.46 ± 0.21	2.12 ± 0.63	2.77 ± 0.48 ^a^	2.43 ± 0.26 ^b^	2.49 ± 0.58	2.36 ± 0.31

**Table 3:** Bone scintigraphic index at week 10 and 22. Comparisons were performed according to the genotype, the influence of CKD, of the diet, and the combination of CKD and diet. Superscript symbols indicate significant difference to the corresponding group, findings in Gα**_q/11_** KO mice significantly different to respective WT mice are given in bold (*p* < 0.05). WT = wild type mice, KO = Gα_q/11_ knockout mice, WT_CKD_ = wild type mice with CKD, KO_CKD_ = Gα_q/11_ knockout mice with CKD, WT_HP_ = wild type mice with high phosphate diet, KO_HP_ = Gα_q/11_ knockout mice with high phosphate diet, WT_CKD-HP_ = wild type mice with CKD and high phosphate diet, KO_CKD-HP_ = Gα_q/11_ knockout mice with CKD and high phosphate diet.

	Scintigraphic Index at Week 10 (Counts·s^−1^·Pixel^−1^·MBq^−1^·g^−1^ × 10^5^)	Scintigraphic Index at Week 22 (Counts·s^−1^·Pixel^−1^·MBq^−1^·g^−1^ × 10^5^)
**WT ^a^**	11.75 ± 0.61 (*n* = 33)	6.47 ± 1.55 (*n* = 9)
**KO ^b^**	14.80 ± 0.70 (*n* = 35) ^a^	16.19 ± 1.63 (*n* = 7)
**WT_CKD_^c^**		6.28 ± 0.83 (*n* = 13) ^a^
**KO_CKD_^d^**		**12.10 ± 1.19 (*n* = 10) ^b,c^**
**WT_HP_^e^**		5.31 ± 1.46 (*n* = 6)
**KO_HP_^f^**		12.00 ± 2.19 (*n* = 8)
**WT_CKD-HP_^g^**		5.60 ± 0.74 (*n* = 7) ^a^
**KO_CKD-HP_^h^**		10.11 ± 1.52 (*n* = 9) ^b^

**Table 4:** Bone parameters from non-decalcified lumbar vertebrae. WT = wild type mice, KO = Gα_q/11_ knockout mice, WT_CKD_ = wild type mice with CKD, KO_CKD_ = Gα_q/11_ knockout mice with CKD, BV/TV = bone volume/tissue volume, BS/BV = bone surface/bone volume, OV/BV = osteoid volume/bone volume, OS/BS = osteoid surface/bone surface, Ob.S/BS = osteoblast surface per bone surface), MAR = mineral apposition rate. *n* = four mice per group; Superscript symbols indicate significant difference to corresponding group, findings in Gα_q/11_-KO mice significantly different to respective WT mice are given in bold (*p* < 0.05).

	WT	KO	WT_CKD_	KO_CKD_
**BV/TV(%)**	26.9 ± 5.5	24.7 ± 4.2	25.9 ± 3.2	32.2 ± 14.6
**BS/BV(%)**	4.0 ± 0.4	4.8 ± 0.5	3.8 ± 0.6	3.6 ± 1.2
**Trabecular Thickness (µm)**	50.2 ± 5.1	42.0 ± 4.4	54.1 ± 7.4	63.3 ± 30.3
**Trabecular Number**	5.3 ± 0.7	5.9 ± 0.5	4.9 ± 0.9	5.1 ± 0.7
**Trabecular Separation (µm)**	139.0 ± 28.0	139.4 ± 28.0	157.9 ± 38.9	134.4 ± 38.8
**OV/BV (%)**	2.8 ± 1.7	4.6 ± 1.4	3.1 ± 2.4	4.2 ± 1.4
**OS/BS(%)**	11.1 ± 4.2	22.1 ± 9.5	14.8 ± 7.4	18.7 ± 3.8
**Ob.S/BS (%)**	23.0 ± 3.0	31.9 ± 8.6	20.8 ± 11.5	29.5 ± 5.5
**MAR (µm/d)**	1.7 ± 0.3	2.1 ± 0.4	1.5 ± 0.3	1.6 ± 0.4
**Mineralizing Surface (%)**	7.0 ± 3.0	12.9 ± 6.6	4.1 ± 3.6	8.5 ± 3.3

## Data Availability

Data supporting reported results can be asked to the authors.
